# Autoinhibited Protein Database: a curated database of autoinhibitory domains and their autoinhibition mechanisms

**DOI:** 10.1093/database/baae085

**Published:** 2024-08-28

**Authors:** Daeahn Cho, Hyang-Mi Lee, Ji Ah Kim, Jae Gwang Song, Su-hee Hwang, Bomi Lee, Jinsil Park, Kha Mong Tran, Jiwon Kim, Phuong Ngoc Lam Vo, Jooeun Bae, Teerapat Pimt, Kangseok Lee, Jörg Gsponer, Hyung Wook Kim, Dokyun Na

**Affiliations:** Department of Biomedical Engineering, Chung-Ang University, Seoul 06974, South Korea; Department of Biomedical Engineering, Chung-Ang University, Seoul 06974, South Korea; Department of Biomedical Engineering, Chung-Ang University, Seoul 06974, South Korea; Department of Bio-integrated Science and Technology, College of Life Sciences, Sejong University, Seoul 05006, Republic of Korea; Department of Biomedical Engineering, Chung-Ang University, Seoul 06974, South Korea; Department of Bio-integrated Science and Technology, College of Life Sciences, Sejong University, Seoul 05006, Republic of Korea; Department of Bio-integrated Science and Technology, College of Life Sciences, Sejong University, Seoul 05006, Republic of Korea; Department of Biomedical Engineering, Chung-Ang University, Seoul 06974, South Korea; Department of Bio-integrated Science and Technology, College of Life Sciences, Sejong University, Seoul 05006, Republic of Korea; Department of Biomedical Engineering, Chung-Ang University, Seoul 06974, South Korea; Department of Bio-integrated Science and Technology, College of Life Sciences, Sejong University, Seoul 05006, Republic of Korea; Department of Biomedical Engineering, Chung-Ang University, Seoul 06974, South Korea; Department of Life Science, Chung-Ang University, Seoul 06974, Republic of Korea; Center for High-Throughput Biology, University of British Columbia, 2125 East Mall, Vancouver, BC V6T 1Z4, Canada; Department of Bio-integrated Science and Technology, College of Life Sciences, Sejong University, Seoul 05006, Republic of Korea; Department of Biomedical Engineering, Chung-Ang University, Seoul 06974, South Korea

## Abstract

Autoinhibition, a crucial allosteric self-regulation mechanism in cell signaling, ensures signal propagation exclusively in the presence of specific molecular inputs. The heightened focus on autoinhibited proteins stems from their implication in human diseases, positioning them as potential causal factors or therapeutic targets. However, the absence of a comprehensive knowledgebase impedes a thorough understanding of their roles and applications in drug discovery. Addressing this gap, we introduce Autoinhibited Protein Database (AiPD), a curated database standardizing information on autoinhibited proteins. AiPD encompasses details on autoinhibitory domains (AIDs), their targets, regulatory mechanisms, experimental validation methods, and implications in diseases, including associated mutations and post-translational modifications. AiPD comprises 698 AIDs from 532 experimentally characterized autoinhibited proteins and 2695 AIDs from their 2096 homologs, which were retrieved from 864 published articles. AiPD also includes 42 520 AIDs of computationally predicted autoinhibited proteins. In addition, AiPD facilitates users in investigating potential AIDs within a query sequence through comparisons with documented autoinhibited proteins. As the inaugural autoinhibited protein repository, AiPD significantly aids researchers studying autoinhibition mechanisms and their alterations in human diseases. It is equally valuable for developing computational models, analyzing allosteric protein regulation, predicting new drug targets, and understanding intervention mechanisms AiPD serves as a valuable resource for diverse researchers, contributing to the understanding and manipulation of autoinhibition in cellular processes.

**Database URL**: http://ssbio.cau.ac.kr/databases/AiPD.

## Introduction

Autoinhibition is a mechanism through which cellular proteins function via self-regulation by transient intramolecular interaction between different parts of the protein [1]. Generally, the interacting parts can be clearly divided into the functional site(s), such as a domain or sequence region that harbors a catalytic site, a binding interface or a cellular localization sequence, and an autoinhibitory domain (AID). AIDs are crucial in the self-regulation of cellular proteins by modulating the activity or impact of the functional site(s) within the same protein [2]. These interactions can vary widely, reflecting the diverse structural forms of AIDs, which range from fully folded domains to entirely intrinsically disordered regions [3]. The presence of an AID ensures that proteins are active only when needed, thereby preventing aberrant or premature activity that could disrupt cellular processes. This regulation is important for maintaining cellular homeostasis and appropriately responding to cellular signals.

Recent advances in experimental techniques have led to the identification of an increasing number of autoinhibited proteins [4–6], alongside a plethora of autoinhibitory mechanisms involving structured and disordered AIDs. Significant research efforts are often required to elucidate the intricate mechanisms through which AIDs affect protein activity [7–10]. At the core of the regulation is always a transition between an active and an inhibited conformation. The transition of the autoinhibited proteins between the active and inhibited states can involve large conformation changes, whereby the AID undergoes significant positional changes and, sometimes, even overall fold changes, or very subtle structural rearrangements [11–15]. The inhibited conformation is the default position of most autoinhibited proteins, while the transition to an active confirmation can be induced by biomolecular partner binding, post-translational modifications (PTMs), or proteolysis of the AID [1]. However, interactions with partners and PTMs can also reinforce or induce autoinhibition, although less frequently.

Since autoinhibition enables the activation of proteins in cells in a controlled and precise manner upon the arrival of appropriate signals, it represents a common regulatory mechanism that is widely found in signaling and metabolic pathways. Moreover, it prevents spurious activation of signaling cascades [1]. Thus, autoinhibition is a present mechanism of regulation in diverse cellular proteins that are involved in gene expression, cell cycle control, and other key cellular processes [16–18]. In a previous computational study, ∼30% of the human proteome was predicted to be autoinhibited [19]. Given the prevalence of autoinhibition and its involvement in key cellular processes, it is clear that maintaining proper autoinhibition of proteins is crucial for cellular homeostasis. Recently, it was found that mutations that disrupt autoinhibition in proteins are implicated in various diseases, such as cancer, neurodegenerative disorders, and autoimmune diseases [20–24]. For instance, AKT1 is a serine–threonine protein kinase that regulates the proliferation and survival of various cell types, and is autoinhibited through an intramolecular interaction between its pleckstrin homology and kinase domain, which can be mitigated by C-tail phosphorylation upon binding to phosphatidylinositol 3,4,5-triphosphate. Many studies have investigated how mutations at the interface between the domains disrupt autoinhibition—in this instance—mutations cause AKT1 to enter a hyperactive state, which is associated with cancer and disorders associated with unregulated cell proliferation [25].

Unsurprisingly, using small molecules to modulate autoinhibition has become an important therapeutic strategy [26, 27]. The restoration of AKT1 autoinhibition using small molecule inhibitors, such as Ipatasertib (GDC-0068), MK-2206, and triciribine has proven highly successful in cancer treatment [28]. Another successfully exploited autoinhibitory mechanism is through Rho-associated protein kinase (ROCK), which is involved in various cellular processes, including cytoskeletal organization and cell contraction. ROCK is autoinhibited by its N-terminal domain, which blocks its kinase domain activity via intramolecular interactions. The drug fasudil binds to the N-terminal domain of ROCK and, thus, disrupts the autoinhibitory interaction, thereby allowing ROCK to become activated [29]. This activation of ROCK has been shown to have therapeutic effects on human diseases, including hypertension, pulmonary hypertension, and stroke [30]. However, it is important to note that while targeting protein autoinhibition can be an effective strategy in the development of drugs, a deep understanding of protein structure and molecular mechanism of autoinhibition in the cellular context is a prerequisite for any such endeavor.

**Figure 1.**
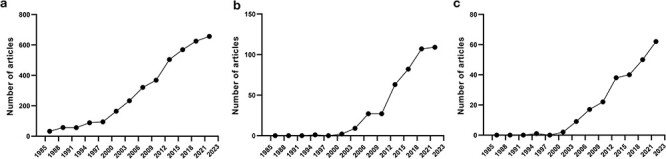
Number of published articles on autoinhibited proteins in PubMed from 1985 to 2023 at 3-year intervals, including articles on autoinhibited proteins (a), autoinhibited proteins + disease (b), and autoinhibited proteins + drugs (c).

The provided examples illustrate that there is a great need to discover autoinhibitory mechanisms in proteins and catalog their modes of action. Therefore, it is unsurprising that there is an extensive number of articles already on autoinhibited proteins (Fig. 1a), their relationship to diseases (Fig. 1b), and drugs that modulate them (Fig. 1c), which is also constantly increasing. Previously, we studied the biochemical properties of different autoinhibited proteins [3, 31] and developed a machine-learning model to predict disordered AIDs [19]. However, the model was trained using a limited number of known autoinhibited proteins. The lack of curated resources that provide access to comprehensive sets of autoinhibited proteins and their AIDs has made it difficult to systematically understand and catalog known autoinhibited mechanisms. Therefore, the therapeutic exploitations of autoinhibition could greatly benefit from a better understanding of these mechanisms.

Here, we introduce the Autoinhibited Protein Database (AiPD), which comprises information on 45 913 AIDs within 45 148 autoinhibited proteins: 698 AIDs were extracted from 532 experimentally characterized autoinhibited proteins across 51 species documented in 864 articles, 2695 AIDs within 2096 autoinhibited proteins across 118 species annotated based on sequence similarity, and 42 520 AIDs within computationally predicted autoinhibited proteins across 2180 species. The AiPD provides diverse annotations for each included autoinhibited protein, such as autoinhibited/relieved structures, domain locations, intrinsic disorder content, PTM sites, functional motif locations, information on sequence variants, and associated diseases, as well as associated GO terms and KEGG pathways. The AiPD also provides a sequence search tool to help users identify and analyze putative AIDs within a query sequence by comparing it with the AIDs previously deposited in the AiPD. To provide an example of how the AiPD can be used for bioinformatic analyses, we analyzed enriched PTMs within the AIDs in the AiPD and discovered that phosphorylation alongside two other PTMs, such as glycosylation and acetylation, was highly enriched, thereby providing insights into a lesser-studied mechanism of autoinhibition regulation and implying that diverse PTMs may be used to provide multilayered regulation for fine-tuning their activity.

We believe that the AiPD will be a valuable resource that can advance our understanding of the significance of autoinhibition in protein activity regulation. It will also aid in pharmaceutical research aimed at developing novel therapeutics for diseases caused by alterations in protein autoinhibition.

## Methods

### Database construction

**Figure 2.**
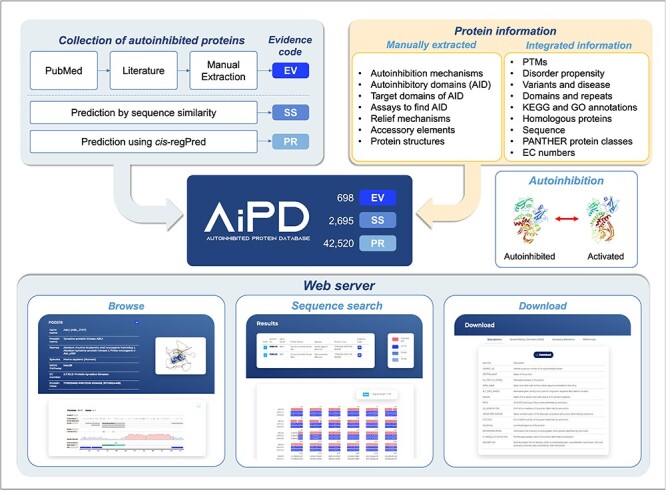
Schematic illustration of the AiPD.

The aim of our database, AiPD, was to provide a comprehensive list of all the currently known proteins that undergo autoinhibition. In addition, we aimed to provide information on numerous features related to the autoinhibited proteins, such as the locations of the AIDs, which intramolecular target domain is regulated by the AIDs, how regulation is achieved and relieved, and which diseases each autoinhibited protein is associated with. Thus, this information can be used to improve our understanding of their function and regulation. To achieve this goal, we extracted information on autoinhibited proteins, their AIDs, and their regulation mechanisms from the literature and complemented it with various informations from accessible databases (Fig. 2).

#### Collection of autoinhibited proteins and AIDs

To collect autoinhibited proteins and their AIDs from the literature, PubMed and Protein Data Bank (PDB) databases were searched for articles and entries on autoinhibited proteins using autoinhibition-related keywords, such as “autoinhibition,” “autoinhibit,” “autoinhibited protein,” “autoregulate,” “autoregulated protein,” “*cis*-regulate,” and “*cis*-regulated protein.” This search covered publications up to February 2024. AIDs within the autoinhibited proteins and their mechanisms of action were manually extracted from the collected articles. During this search, we encountered instances of inter-molecular autoinhibition between subunits of protein complexes, such as multimeric plasma membrane ATPases [32] and the multimeric protein kinase CK2 [33]. As the definition of autoinhibition in AiPD is an intra-molecular regulation within a polypeptide and our primary focus was on intra-molecular autoinhibition, the current version of AiPD included only intra-molecularly autoinhibited proteins. Notably, certain ATPases (e.g. ATPase 2 and its UniProt accession number is P19456) exhibit both inter- and intra-molecular autoinhibition [34], and these were included in our database due to the intra-molecular autoinhibition.

In addition to experimentally identified AIDs, we also included potential AIDs through sequence comparison or computational prediction. For sequence comparison, human, rat, and mouse proteins homologous to the experimentally identified autoinhibited proteins were obtained from the Homologous Gene Database (HGD) [35]. If the sequence identity between an experimentally confirmed AID and an aligned region in a homolog was >50%, the homologous protein and its putative AID were also included in the AiPD. For computational predictions, proteins deposited in UniProt were queried against *cis*-regPred, and potential AIDs were extracted. *cis*-regPred is a computational method that we developed to identify intrinsically disordered protein regions, which could act as sequence elements that regulate protein activity via *cis* interactions [19]*. cis*-regPred exploits various features that we found enriched in AIDs, such as phosphorylation clusters, alternative splicing sites, high charge density, and electrostatic complementarity with the inhibited target domain/region. *cis*-regPred has been extensively tested and validated using independent test sets [19]. In summary, there are three types of AIDs in the AiPD: “Experimentally validated” (EV), “annotated by sequence similarity” (SS), and “predicted by cis-regPred” (PR). Consequently, the AiPD contains 698 EV-assigned AIDs within 532 autoinhibited proteins, 2695 SS-assigned AIDs within 2096 proteins, and 42 520 PR-assigned AIDs within the proteins from 2180 species.

The details of the compiled information in the AiPD are summarized in Table 1. In the next sections, we briefly describe the key information contained in Table 1 and the collection process.

**Table 1.** Autoinhibited protein information extracted from the literature and other databases and integrated into the AiPD

Category	Subcategories	Items	Description
Protein name			The common and alternative names of the autoinhibited protein.
UniProt AC			UniProt accession number of the autoinhibited protein.
Description			The function of the autoinhibited protein, known AIDs and target domain, experimental methods used to identify AIDs, and autoinhibition and relief mechanism are described.
AIDs			Domains or sequence segments that modulate the activity of the *cis* target domain. The boundary of AIDs mentioned in the literature was adopted in the AiPD since there are no agreed boundary definitions. If two or more boundaries have been reported in the literature, the narrowest boundary was assigned to the AID.
	Target domains		Domain or region regulated by the AID.
	Evidence codes		Evidence code explaining how an AID was identified. Each AID has at least one evidence code.
		EV	AID that is annotated with this code was identified experimentally. The experimental method is specifically defined in the “Assay” category.
		SS	AID that is annotated with this code was identified by sequence comparison with AIDs within homologous proteins.
		PR	AID that is annotated with this code was predicted using the disordered AID prediction model *cis*-regPred.
	Assays		Experimental method used to identify AIDs. This is available only when the AID is annotated with EV.
		Deletion assay	If deletion or truncation of a region enhances protein activity, the deleted region is assigned as the AID.
		Mutagenesis experiment	If residue modifications alter protein activity, the residue or the conserved motif containing the mutated residue is assigned to the AID.
		Split protein assay	AID and target domain were split from a protein, and their interaction (complex formation) in *trans* was experimentally confirmed.
		Peptide inhibitor test	If a protein is inhibited by a peptide and the same peptide sequence exists within the protein, the peptide sequence is assigned with the AID.
		Structural analysis	AIDs were identified by comparing active and inactive protein conformations, which were determined by X-ray crystallography or other biophysical methods.
	Relief mechanisms		Autoinhibition relief mechanism for each AID, if available in the literature.
		PTM	When the residue(s) is/are post-translationally modified (PTM) via phosphorylation, glycosylation, etc., the activity of the target domain is recovered.
		Partner binding	Autoinhibition is relieved by the binding of a partner protein. The partner protein can bind to the AID directly or other protein regions, resulting in protein activation
		Ligand binding	Similar to “partner binding,” autoinhibition is relieved by the ligand binding, e.g. Ca^2+^.
		Cleavage	Autoinhibition is relieved by cleavage of the AID by proteases.
		Others	“Others” denotes a relief mechanism that is inconsistent with any of the above criteria.
Accessory elements			Accessory element is a region involved in autoinhibition but does not make direct contact with a target domain or is a regulatory region within a catalytic domain, such as an activation loop within the kinase domain.
Structures			If structures are available in PDB, autoinhibited and relieved structures are included AiPD.
Phosphorylation and glycosylation sites			Phosphorylation sites and N-linked glycosylation sites obtained from PhosphoSitePlus and *N*-GlycositeAtlas, respectively.
Variants and diseases			Residue variants of autoinhibited proteins and their associated diseases.
Domains and motifs			Domains and functional sites obtained from InterPro.
GO annotations			GO terms assigned to autoinhibited protein, which were obtained from UniProt.
Homologous proteins			A list of homologous proteins for an autoinhibited protein, obtained from the HGD database.

#### Description of autoinhibited proteins

The function of autoinhibited proteins, their AIDs and target domains, autoinhibition mechanism and relief mechanism, and the experiments used to identify the AIDs are summarized in the free text under “Description” section (Table 1).

#### Target domains

In the AiPD, the target domain is defined as a domain that is inhibited by AIDs. Many of these target domains are catalytic domains, such as kinase domains. However, non-catalytic functional segments can also be regulated by AIDs, such as different types of protein–protein or protein–DNA interaction domains, or sequence regions. Therefore, we used the term “target domain” in the AiPD instead of “target catalytic domain” to avoid any bias and misguidance.

#### Autoinhibitory domains

The AiPD contains three types of AIDs: EV, SS, or PR [19], as described in “Collection of autoinhibited proteins and AIDs” section. For EV AIDs, we collected additional information from the literature on the experimental assays used to identify the AIDs as well as information on the autoinhibition relief mechanism.

We classified the assays used to identify the AIDs into five groups: deletion assay, mutagenesis experiment, split protein assay, peptide inhibitor test, and structural analysis. “Deletion assay” indicates that deletion or truncation of an AID was used to demonstrate the relief of autoinhibition. “Mutagenesis experiments” indicate that the mutations within the AIDs were used to probe changes in protein activity. “Split protein assay” indicates that AIDs and target domains were separately expressed to test inhibition of function via *trans* interaction. The “peptide inhibitor test” indicates that a peptide inhibitor homologous to the AID was used to test for *trans* inhibition. “Structural analysis” indicates that the AID was identified by comparing the available structures for the autoinhibited and active protein conformations.

We classified autoinhibition relief mechanisms into five groups: “PTM,” “partner binding,” “ligand binding,” “cleavage,” and others. “PTM” indicates that the activity of the target domain is modulated via PTMs of the autoinhibited protein, such as phosphorylation, glycosylation, and/or acetylation. “Partner binding” indicates that autoinhibition is modulated through binding a partner protein. The partner protein can bind to the AID directly or to other regions of the autoinhibited proteins, resulting in a structural change that activates/inhibits the protein. “Ligand binding” indicates that similar to partner binding, autoinhibition is modulated by a ligand binding, e.g. Ca^2+^. “Cleavage” indicates that autoinhibition is modulated (mostly relieved) by the AID being cleaved from the rest of the protein. Though zymogens are also activated by the proteolysis of their inhibitory parts, they were not included in the AiPD. “Others” indicate a relief mechanism that is different from the other categories. All this information was carefully collected and curated from the original publications describing the autoinhibited proteins.

#### Accessory elements

Accessory elements are the regions that are involved in autoinhibition but do not make direct contact with the target domains or are regulatory regions within a catalytic domain, e.g. the activation loops within kinase domains. Accessory elements were obtained from the literature. The activation loops were obtained from the literature as well as InterPro [36].

#### Structures

Certain autoinhibitory domains have been identified through the analysis of protein structures in both autoinhibited and relieved states. When such structures are available in the PDB [37], they are incorporated into AiPD as representative structures for both states. Additionally, other structures found in the PDB are included in AiPD. Given that not all protein structures have been resolved, predicted structures from the AlphaFold Protein Structure Database have also been incorporated into AiPD to enhance our understanding [38].

#### Post-translational modification sites

Since PTMs frequently play a key role in autoinhibition regulation, we also included phosphorylation and glycosylation sites in the AiPD. This information was collected from PhosphoSitePlus [39] and *N*-GlycositeAtlas [40].

#### Protein classes

Proteins deposited in AiPD were categorized according to the functional classes defined in the PATHER database, a protein database used for classification and analysis of protein sequences and their functions [41]. Examples of the PANTHER protein class include scaffold/adaptor protein, non-receptor tyrosine protein kinase, cytoskeletal protein, etc.

#### Others

The malfunction of autoinhibited protein has been associated with diverse diseases. Thus, we included information on amino acid variations and diseases associated with autoinhibited proteins; data were also collected from UniProt [42]. Since both the structured domains and intrinsically disordered regions can be involved in autoinhibitory regulation [43], we included disorder propensities of autoinhibited proteins in the AiPD by predicting the disordered regions using IUPred3 [44]. GO annotations from Gene Ontology [45] were also included in the AiPD to provide more systematic information on the functions of autoinhibited proteins.

### Web server construction

The AiPD runs on Ubuntu, which is a Linux-based web server. The backend of the AiPD is run on Python 3.8.5 and Flask. The interactive web interface was developed using HTML5, CSS, Bootstrap (version 4.2.1), JavaScript, and JQuery (version 1.11.1). Web server sequence search functionality was implemented using USEARCH (version 11.0.667) [46] and ClustalW2 [47]. The feature viewer was implemented based on the neXtProt feature viewer in JavaScript [48].

### Analysis of PTM enrichment within AIDs

For PTM enrichment analysis, AIDs of human autoinhibited proteins with an annotation of EV were collected from the AiPD. Human-reviewed proteins previously deposited in UniProt were also collected and used as a reference. Similar sequences were discarded by USEARCH following a cutoff identity of 0.6 [46]. PTM information was obtained from dbPTM [49]. Enriched PTMs were identified by Fisher’s exact test. Human autoinhibited proteins containing a particular PTM within their AIDs were collected for KEGG pathway enrichment analysis. Human proteins in UniProt containing a PTM within their sequence were also collected as a reference. Using those proteins, KEGG pathway enrichment analysis was performed using DAVID [50].

## Results and discussion

**Figure 3.**
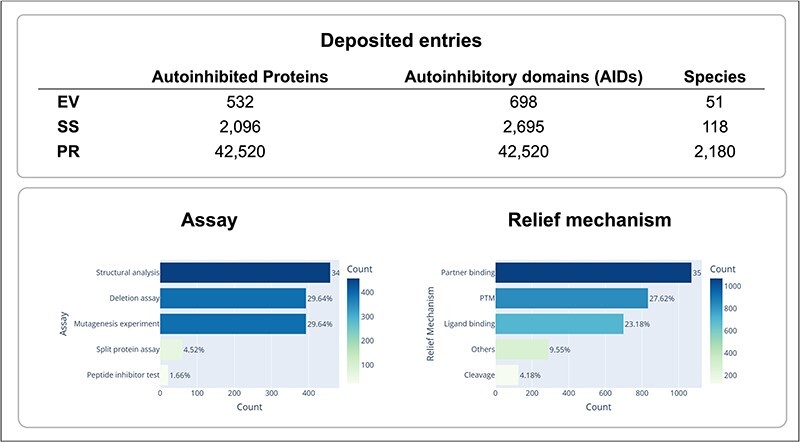
Statistics of deposited autoinhibited proteins and their regulation mechanisms.

Experimental methods used to identify autoinhibited proteins and their AIDs, and determine their boundaries, as well as their regulation mechanisms are very diverse. However, at the core of all these experiments is an assay that demonstrates the alterations in each AID as either deletions or mutations that enhance protein activity. Depending on the specific assay that was used, the identified AIDs can range in length from just a few residues to hundreds of residues. A large truncation assay can only be used to identify an approximate region containing the AID, whereas a residue-specific mutagenesis assay can be performed to find a specific residue, which is critical for autoinhibition. In addition, structural features of AIDs and their mechanisms of action can be very diverse. Certain AIDs are structural domains, while other AIDs are intrinsically disordered. Though many AIDs sterically mask the active or binding site of a target domain, certain AIDs inhibit function allosterically. Given this diversity in autoinhibited proteins and their AIDs, we aimed to provide pictures of the proteins that are as complete as possible by providing information on various aspects of these autoinhibited proteins and their AIDs, including the identification of the AIDs, their locations in the protein sequence, the domains or regions they target, and the mechanisms through which they evoke and halt their inhibitory functions. The information we collected is summarized in Fig. 2 and Table 1. In the AiPD, there are three types of AIDs: EV, SS, and PR. Consequently, the AiPD contains 698 EV-assigned AIDs within 532 autoinhibited proteins, 2695 SS-assigned AIDs within 2096 proteins, and 42 520 PR-assigned AIDs within the proteins from 2180 species (Fig. 3).

### Web server

**Figure 4.**
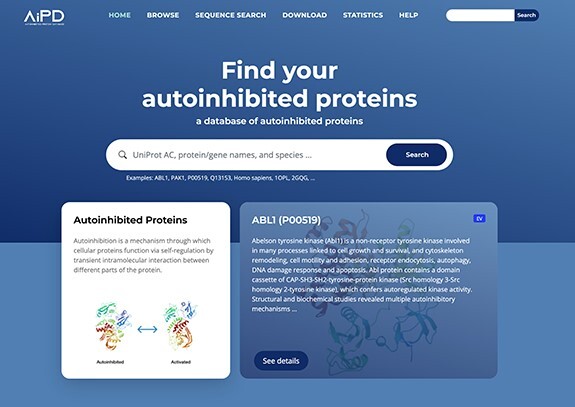
Web server of AiPD.

**Figure 5.**
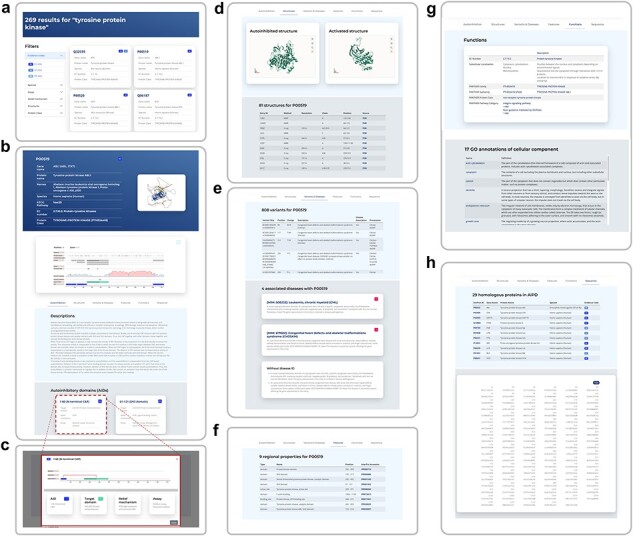
A web page example for human ABL1, showing the search result by “protein tyrosine kinase” (a), a detailed information web page on human ABL1 and its autoinhibition (b), a schematic diagram of autoinhibition with an AID and its target (c), representative structures of ABL1 in autoinhibited and relieved states (d), sequence variants and their associated diseases (e), regional protein properties, such as domains and motifs (f), EC number, PANTHER protein class, and GO annotations of ABL1 (g), and the canonical sequence of ABL1 and its homologous proteins deposited in AiPD (h).

The AiPD web server is shown in Fig. 4, the features that our AiPD provides with an example are described below (Fig. 5).

#### Search and view

To the best of our knowledge, the AiPD is the first database for autoinhibited proteins and their AIDs, which was developed to provide comprehensive views on this important group of proteins. The web server provides an interactive interface that helps the user intuitively explore and extract the diverse information related to each autoinhibited protein.

An example of how to use and navigate through the AiPD is provided in Fig. 5. The user can enter any keyword or information in the search bar, for example, the protein’s UniProt accession number, its name, or PDB code. In the AiPD, we allocated each UniProt accession number to its corresponding autoinhibited protein, utilizing it as the primary ID to circumvent the ID inconsistency issues frequently encountered in bioinformatics field. In the example provided, we conducted a search for “tyrosine protein kinase.” The identified proteins are listed by their gene names, accompanied by evidence code (Fig. 5a). Autoinhibited proteins containing experimentally identified AIDs (annotated with EV) appear first followed by those containing potential AIDs (annotated with SS or PR). The listed proteins that share the same evidence code are further sorted by gene name.

Clicking on the UniProt accession number opens the web page of the selected autoinhibited protein. In the example provided, the human “tyrosine protein kinase ABL1” (P00519) was chosen (Fig. 5b–h). At the top of the protein page, a brief summary of the protein is provided, which includes UniProt accession number, gene names, protein names, species of origin, KEGG pathway, EC number, and PANTHER protein classes to which the protein belongs.

In the “Description” section under “Autoinhibition” tab, information is provided on how autoinhibition is achieved and relieved, which domains or regions are involved in the regulation, and how AIDs were experimentally or computationally identified in the free text (Fig. 5b). The description was carefully written by experts after reading the literature to ensure the information provided was reliable. References are also listed at the end of the section to provide additional reading and information. For the autoinhibited proteins identified by sequence similarity with their homologous proteins, their descriptions were adopted from the homologous proteins. In Fig. 5b, a user can identify five AIDs (CAP, SH3, SH2, a linker region, and F-actin binding domain), which are involved in autoinhibiting the ABL1 tyrosine protein kinase domain (target domain) and that autoinhibition is relieved by PTMs of CAP or the linker region acting as AID, or by F-actin binding to the F-acting binding domain. Through the interactive view, a user can easily explore the data provided by zooming in and out, which helps provide clearer information on the specific elements involved in autoinhibition, such as the exact locations of the AIDs or PTM sites. Clicking the name of an AID under the “Autoinhibitory domain (AID)” section results in a new window opening that exhibits an intuitive relationship between an AID and its target domain (Fig. 5c).

Indeed, phosphorylation and glycosylation sites are also displayed in the interactive view. If these sites are within an AID, they may be involved in regulating autoinhibition. There are many phosphorylation sites within the AIDs of ABL1 and one N-linked glycosylation site within the SH2 domain (Fig. 5b). For example, the phosphorylation of tyrosine residues within the linker region, which serves as the AID, has been found to cause its dissociation from the tyrosine protein kinase. This event opens the active site of the tyrosine protein kinase domain, relieving autoinhibition [51]. The N-linked glycosylation site within the SH2 domain has not previously been studied in the context of autoinhibition, and our database entry may suggest a putative role of glycosylation in autoinhibition regulation.

In “Structures” tab, the representative structures of the autoinhibited protein in autoinhibited and relieved states are displayed to facilitate the user to get deep insight into the autoinhibition mechanism (Fig. 5d). Protein structures were obtained from PDB if available. Given that not all protein structures have been resolved, predicted structures from the AlphaFold Protein Structure Database have also been incorporated into AiPD to enhance the user’s understanding.

From the “Variants” and “Diseases” tab, a user can determine the genetic variants within the ABL1 AIDs that are associated with diverse diseases, including congenital heart defects, skeletal malformations syndrome, and chronic myelogenous leukemia (Fig. 5e). For intuitive exploration, variants are provided with an interactive view, in which sequence, variants, and AIDs are displayed.

The “Features” tab displays functional sites, such as the presence of domains and motifs in the autoinhibited protein (Fig. 5f). The “GO Annotations” tab shows a list of GO annotations assigned to the autoinhibited protein, which are grouped into three categories: cellular component, molecular function, and biological process. Each GO annotation is linked to the AmiGO2 in the Gene Ontology, thereby allowing for online access to GO terms and the graphical hierarchical structure that the terms belong to (Fig. 5g). The “Sequence” tab shows the canonical sequences of the autoinhibited proteins, which in our example is for ABL1. As similar sequences may possess similar AIDs, homologous proteins with their evidence code are listed, if available (Fig. 5h).

#### Sequence search

**Figure 6.**
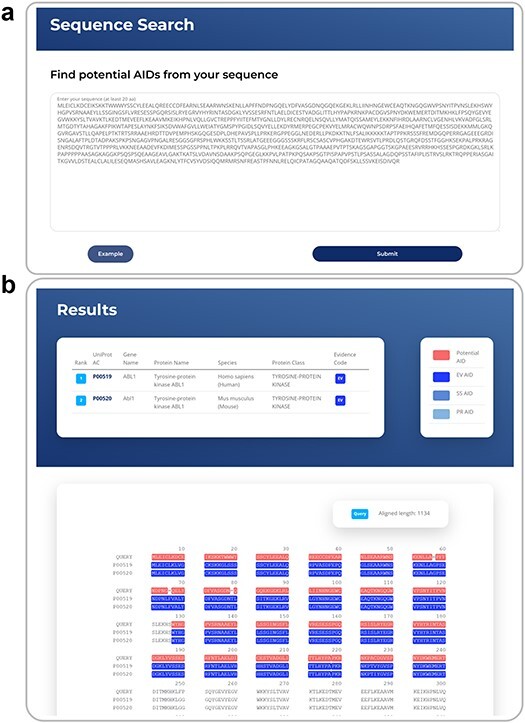
An example of sequence search, showing a case example of sequence search (a) and an exemplar sequence search result with known AIDs highlighted and the corresponding region within a query sequence as a putative AID (b).

In addition to the keyword-based search, the AiPD also provides functionality to search for putative AIDs through the alignment of the user’s query sequence with any autoinhibited protein sequences in the AiPD (Fig. 6a). The user can access this tool via the “Sequence Search” menu. Briefly, the AiPD searches for proteins that are homologous to the user’s query sequence. In the search, only autoinhibited proteins containing experimentally validated AIDs (evidence code of EV) are considered. The sequence-based search is conducted using USEARCH, a tool similar to Blast but faster [46]. When similar sequences are identified up to three, the query sequence along with the identified sequences are aligned and displayed with the AIDs, deposited in the AiPD, highlighted differently depending on their evidence code, since an autoinhibited protein may have multiple AIDs with different evidence codes. This search result enables the intuitive identification of putative AIDs within the user’s query sequence. The multiple sequence alignment is performed by ClustalW2 [47]. In the example provided (Fig. 6b), human ABL1 and murine Abl1 are identified as homologous proteins and aligned with the user’s sequence. The red regions within the user’s query sequence are putative AIDs based on the known AID sequences.

#### Download

The aim of AiPD is to provide both an integrated view of autoinhibited proteins as well as well-formatted data for bioinformatic analyses. Thus, the entire database is available for download on the “Download” page. On this page, a user can also find a description of the file format.

### Exemplar statistical analysis with AIDs in the AiPD

Well-formatted data are a valuable asset in bioinformatic analyses. As an example, we performed the first statistical analysis on the data in the AiPD to gain insights into autoinhibited proteins.

#### Enrichment of PTMs within AIDs

PTMs can serve as a modulator of protein autoinhibition by inducing conformational changes. PTMs are reversible chemical modifications, which allow autoinhibited proteins to switch between a state of “on” and “off” or to fine-tune their activity. The reversible nature of PTMs is essential for regulating protein activity in response to changing cellular conditions.

**Figure 7.**
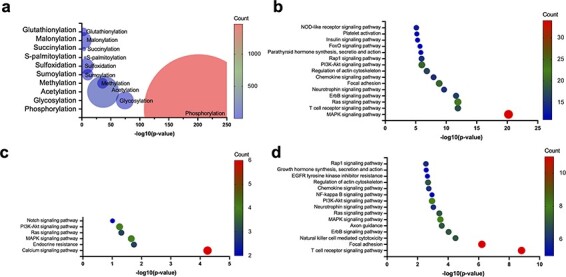
Enrichment of PTMs within the AIDs of human autoinhibited proteins, including enriched PTMs (a) and enriched KEGG pathways within the human autoinhibited proteins containing phosphorylation (b), glycosylation (c), and acetylation (d) sites within their AIDs.

To explore the PTMs that are enriched within the AIDs of autoinhibited proteins, we collected 275 human autoinhibited proteins from the AiPD with an annotation of EV and identified any enriched PTMs within the AIDs in those proteins. As shown in Fig. 7a, phosphorylation exhibits the most significant enrichment within the AIDs in the autoinhibited proteins, which has already been acknowledged as crucial in regulating autoinhibition, particularly in kinase signaling cascades [3, 52, 53]. Furthermore, a spectrum of additional PTMs, including glycosylation, acetylation, methylation, and others, can also exert diverse effects on protein autoinhibition within distinct cellular pathways. Consequently, the presence of aberrant PTMs that occur to autoinhibited proteins can stand as a pivotal hallmark in numerous disease pathologies, spanning various cancer types as well as immune and neurodegenerative disorders [15, 54–57].

Though phosphorylation accounts for most PTMs within the AIDs of the collected human autoinhibited proteins, glycosylation, acetylation, and other PTMs also account for a significant portion of the PTMs within the AIDs. Thus, we also investigated the KEGG pathway enrichment within the human autoinhibited proteins with PTMs in their AIDs. As shown in Fig. 7b–d, a significant portion of phosphorylation, glycosylation, and acetylation events is associated with similar signaling processes, which suggests a wide utility of PTMs within numerous cellular processes. For example, the common pathways are involved in the activation of the Ras protein and the subsequent activation of the MAPK cascade. The Ras–MAPK pathway serves as a central hub that coordinates multiple cellular functions, including cell proliferation and differentiation, apoptosis, immune response, and neuronal plasticity [58–64]. The wide utilization of various PTMs to regulate cellular signaling processes may indicate that the PTMs provide multiple layers of regulation and allow for the precise control of signaling. However, it is important to exercise caution as the research emphasis on autoinhibition may exhibit a bias toward signaling proteins. Consequently, further investigation is required in the future.

#### A case study on the top three PTMs as regulatory mechanisms in autoinhibition

Phosphorylation is one of the most, well-studied PTMs involved in protein autoinhibition [3, 51–53, 65–67]. Catalytic phosphorylation at specific sites by protein kinases has the potential to elicit conformational alterations, which influence the functional propensity of a protein. On the other hand, protein phosphatases catalyze the removal of phosphate groups, thereby restoring the protein to its native state. This reversible process is a ubiquitous feature across a wide array of signaling pathways to regulate the activity of signaling proteins [68–70].

Recently, advanced proteomics methods have successfully identified less studied PTMs. We analyzed the literature to confirm whether other PTMs are also utilized for autoinhibitory regulation and found that glycosylation of AIDs has been shown to contribute to autoinhibition regulation. For example, autoinhibition of epidermal growth factor receptor (EGFR) is achieved via an intramolecular interaction between two domains (domains II and IV). Glycosylation of Asn578 in domain IV induces autoinhibition, whereas the mutation of Asn578Gln disrupts autoinhibition due to the loss of glycosylation [71]. On the contrary, glycosylation of Asn196 in domain II or Asn361 near the C-terminus of domain II reduces autoinhibition [72].

An illustrative case of acetylation-induced functional alteration in an autoinhibited protein is human ETS translocation variant 4 (ETV4), which is a transcriptional activator that plays a vital role in keratinocyte differentiation and is often overexpressed in prostate cancer [15]. Lysine acetylation within the N-terminal AID alleviates autoinhibition by disrupting intramolecular interactions, thereby facilitating DNA binding activation [15].

Additionally, various PTMs can act on the same protein concomitantly to regulate its autoinhibition, which may add another layer of complexity to the precise regulation of protein activity. For instance, the tumor suppressor protein p53 undergoes multiple PTMs to regulate its own activity. The acetylation of specific lysine residues within the C-terminus of p53 can disrupt the autoinhibition and activate p53, allowing it to function as a transcription factor and regulate genes involved in cell cycle control and DNA repair [54]. Likewise, the phosphorylation status of Thr55 in the N-terminal domain of p53 modulates the intramolecular interactions and regulates the DNA-binding affinity of p53 [73]. Similarly, multiple PTMs can have synergistic or opposing effects on autoinhibition depending on the context.

In summary, while the specific mechanisms through which glycosylation and acetylation contribute to the regulation of autoinhibition are not fully understood for all autoinhibited proteins with these PTMs, our simple analysis suggests that these mechanisms in autoinhibition regulation may be more prevalent than previously assumed, although still occur less frequently than phosphorylation.

## Perspectives

Due to the association of autoinhibited proteins with diseases and their critical roles in cellular signaling, autoinhibited proteins are receiving increasing interest. However, there has been no reliable database for autoinhibited proteins thus far. Therefore, we constructed a database for autoinhibited proteins and their regulatory domains (AiPD). The AiPD provides high-quality annotations of autoinhibited proteins and their AIDs, which can help researchers elucidate the roles and mechanisms of interest for autoinhibition and identify potential therapeutic targets. We also constructed a web server that facilitates the search and exploitation of data deposited in the AiPD. Our database will provide a useful resource for researchers studying the mechanism of autoinhibition or developing computational models to predict new autoinhibited proteins and their regulatory domains, and those studying diseases and developing potential drug candidates that alter protein autoinhibition. To keep pace with increasing publications on autoinhibited proteins, the server will be regularly updated, and we are committed to making the AiPD a useful repository for research communities in diverse fields.

## Data Availability

All the datasets generated in this study are available at http://ssbio.cau.ac.kr/databases/aipd.
